# Recent Legislation

**Published:** 1918-11

**Authors:** 


**A Monthly Review of Medicine and Surgery**

EDITOR AND PUBLISHER, DR. A. L. BENEDICT, 228 Summer St., corner of Elmwood Ave., Buffalo, (Address for all communications. Please make personal and telephone calls before 1 P. M.)

Third, in age, on the western continent. Independent, but supports professional organizations. News limited mainly to Western N. Y. and Penn.

Subscription $2.00 a year; $3.00 for two years in advance. Changes and errors in address or failure or duplication of delivery, should be reported immediately.

Yearly Volume 74 NOVEMBER, 1918 Number 4

### Recent Legislation.

Public Health Laws are being enacted by most of the states to meet new conditions affecting public health that increasingly arise in our complex and congested centers of population as well as in rural communities, where such maladies as Spanish Influenza sweep over great cantonments or as small pox over sparsely settled districts.

In order to prevent contagion from the spread of infectious diseases, quarantine laws are enacted and suspects are isolated until the danger of infection is passed.

Both the Federal and State Governments exercise their sovereign powers to preserve the public health. Since the enactment of the Food and Drugs Act of June 30th, 1906, the Congress of the United States has supplemented that statute in the enactment of several amendments thereto. Among other things the Act authorized the secretaries of the Treasury, Agriculture and Commerce and Labor "to make uniform rules and enact regulations to carry out the statute," which the Supreme Court of the United States in the case of U. S. vs. Antikamnia Chemical Company decided might be done. Such power is at least of doubtful propriety in that the violation of any such rule or regulation subjects the person so violating it to prosecution.

The inadvisability of clothing administrative officers with the power "to make uniform rules and enact regulations" as the statute denominates it, which rules and regulations have the same force as the most carefully prepared law, though they may not have any such consideration, as that given to bills in their passage through the committees of and through Congress itself, must be apparent to the Medical Profession as it is to lawyers. Any ambiguity in the phrasing of any such rule or regulation might also subject any member of the profession, failing to correctly interpret any such rule or regulation as it was intended to be interpreted to prosecution. The power "to enact" is a legislative power and under the constitution of the United States and of the states is vested in their legislative departments, where it should be exclusively exercised and not conferred upon executive officers nor upon commissions. The object and purposes of the Food and Drugs Act of June 30th, 1916, are commendable, but its interpretation has been perplexing and expensive, as the records in and out of courts clearly show.

On December 14th, 1914, Congress passed the so-called "Harrison Act," of which the following is the title, namely "An Act to Provide for the Registration of, with Collectors of Internal Revenue, and to impose a Special Tax upon all persons who produce, import, manufacture, compound, deal in, dispense, sell, distribute, or give away opium or coca leaves, their salts, derivatives, or preparations and for other purposes."

That Act makes it unlawful for any person, who has not registered or paid the Special Tax imposed by that act, to have in his possession, or control opium, coca leaves, their salts, derivatives or preparations and it is also declared unlawful for any person to sell, or give away any such drugs without a written order, provided for, except deliveries by physicians to their patients, or on their order and in certain other cases.

In the U. S. v. Fin Fuey Moy 241 U. S. 394, the Supreme Court of the United States in construing one of the Sections of that Act said: "If opium is produced in any of the states obviously the greatest question of power would be raised by any attempt of Congress to make possession of such opium a crime." That indicates the attitude of the Court on that subject and it sustained the lower Courts in discharging an alleged offender of that act. That statute exempts the possession of such drugs prescribed in good faith by physicians and in some other cases.

The foregoing statutes indicate the trend of recent Congressional Legislation, which has given rise to much discussion in medical circles and it now appears that the Federal Registration Act if ever squarely tested in the Supreme Court of the United States, may be declared unconstitutional in some of its sections.

The Harrison Act has been followed by four legislative acts in New York, namely, chapter 363 of the laws of 1914, chapter 327 of the laws of 1915, chapter 431 of the laws of 1917, and chapter 639 of the laws of 1918.

The first three of these legislative acts of the state of New York, enumerate and regulate the use of Habit Forming Drugs. They are repealed by chapter 639 of the laws of 1918, but the repeal does not take effect until February, 1919, except that new sections 421, 422 and 423 of Public Health Law, comprised in the new "Article XXII," added by chapter 639 of the laws of 1918 as to the creation of the Department of Narcotic Drug Control, Commissioner Deputies, and Registry take effect on November 1st, 1918.

This new act is a complete revision of the present laws in relation to narcotic drugs, their sale, dispensation and use and comprises 25 sections, constituting a new Article XXII of the Public Health Law. Section 423 is of present interest to the persons and institutions mentioned therein and reads as follows: "Section 423. Acts prohibited; registry. No person shall possess, sell, distribute, administer or dispense cocaine or opium or its derivatives except as expressly and specifically authorized by the provisions of this article and any unauthorized possession, sale, distribution, administration or dispensation of such drugs is hereby declared to be dangerous to the public health and a menace to the public welfare. No manufacturer, wholesaler, apothecary, physician, dentist, veterinarian or private hospital, sanatorium or institution maintained or conducted in whole or in part for the treatment of disability or disease or inebriety or drug addiction shall purchase, receive, possess, sell, distribute, prescribe, administer or dispense cocaine or opium or its derivatives unless prior thereto he shall have registered with the department his name or style, place of residence and place or places where such business is to be carried on, and received from the department a certificate authorizing him to carry on such business. During the month of January after this article takes effect he shall so register with the department. During each month of June, thereafter he shall, in like manner, register with the department and for such second and each subsequent registry shall pay to the department a fee of one dollar."

The entire article must be read by the profession to understand all its provisions, but enough has been stated herein to put the medical profession on guard, which is all that is attempted in this article. This recent statute must be read in conjunction with the Harrison Act, still in force.

